# Radiation Reveal: Moving from research engagement to involvement

**DOI:** 10.1038/s41416-024-02648-0

**Published:** 2024-04-13

**Authors:** Lisa Whittaker, Jamie A. Dean, Catarina Veiga, Sophie Langdon, Rebecca Drake, Daniel Taylor, Myfanwy-Cerys Williams, Holly Masters, Alex Britton, Mia Cumbo, Nicole Burdis, Kate Mason, Gemma Fay, Emma Smith, Sam Benson, Alfie Halil, Sophie Lambert, Mark N. Gaze, Jenny Gains, Bella Spencer, Alice Taylor-Gee, Samantha Y. A. Terry

**Affiliations:** 1 Wellcome/EPSRC Centre for Medical Engineering, King’s College London, London, UK; 2 Cancer Research UK RadNet City of London, London, UK; 3 Department of Medical Physics and Biomedical Engineering, University College London, London, UK; 4 Institute for the Physics of Living Systems, University College London, London, UK; 5 School of Biomedical Engineering and Imaging Sciences, King’s College London, London, UK; 6 Barts Cancer Institute, Queen Mary University London, London, UK; 7 Wellcome/EPSRC Centre for Interventional and Surgical Sciences (WEISS), University College London, London, UK; 8 Centre of the Cell, Queen Mary University of London, London, UK; 9 Young Adult Participants, London, UK; 10 University College London Hospitals NHS Foundation Trust, London, UK

**Keywords:** Education, Lab life

## Abstract

Here, we report on the process of a highly impactful and successful creative, collaborative, and multi-partner public engagement project, Radiation Reveal. It brought together ten young adults aged 17–25-year-olds with experience of radiotherapy with researchers at Cancer Research UK RadNet City of London across three 2-hour online workshops. Our aims were to 1) initiate discussions between young adults and radiation researchers, and 2) identify what people wish they had known about radiotherapy before or during treatment. These aims were surpassed; other benefits included peer support, participants’ continued involvement in subsequent engagement projects, lasting friendships, creation of support groups for others, and creation and national dissemination of top ten tips for medical professionals and social media resources. A key learning was that this project required a dedicated and (com)passionate person with connections to national cancer charities. When designing the project, constant feedback is also needed from charities and young adults with and without radiotherapy experience. Finally, visually capturing discussions and keeping the door open beyond workshops further enhanced impact. Here, we hope to inform and inspire people to help project the patient voice in all we do.

## Rationale

Radiation Reveal brought ten young adults with radiotherapy experience together with researchers. The rationale for the project came from a desire to carry out impactful patient and public involvement, beyond  public engagement of the inspiring and informing type, that influences radiation research. Radiation Reveal moved our experience from public engagement (focusing on raising awareness, sharing research knowledge and findings) towards involvement (working collaboratively with patients and the public and sharing decision-making and result dissemination). This project was carried out within Cancer Research UK RadNet City of London (CoL) across King’s College London, University College London, and Queen Mary University of London (QMUL). The first step was to ascertain who we needed to engage with.

The Brightlight programme, which carries out research with teenagers and young adults to improve their experience and outcome after a cancer diagnosis, highlighted the unique needs of young people with cancer [1]. Having cancer as a teenager or young adult (TYA) is different from having it as a child or older adult [2]. For example, a cancer diagnosis during that period affects a critical and complex stage of life development, disrupting physical health, social and educational goals, and psychological well-being [1, 3]. Also, long-term treatment effects include increased risks for cardiovascular disease, diabetes, obesity, fertility impairment and second cancers. TYAs are underrepresented in clinical trials, which limits relevant treatment knowledge [4]. Finally, cancer services are tailored towards children and older adults; young people fall into ‘the grey zone’ or ‘no man’s land’ [1].

Radiotherapy, a critical component of many treatment pathways, is often known and understood less than chemotherapy, and patients’ experiences vary. Understanding these experiences is key to improving patients’ care and outcomes [5]. After several rounds of discussions between researchers and clinical oncologists across RadNet CoL and the Centre of the Cell at QMUL, we realised the voice of TYAs experiencing radiotherapy was missing; few prior studies have focused on this patient group [6]. As such, we sought to connect with 17–25-year-olds with experience of radiotherapy and initiate discussions between them and radiation researchers, in the hope of influencing research and identifying what people wish they had known about radiotherapy before or during treatment. Our considerations were later vindicated with a participant describing their radiotherapy experiences as:

*“It was a nightmare […] I just felt different from my friends because they got proper jobs. And started getting houses and stuff and I was like two years behind them […] When I was in the hospital, I was quite isolated. Especially with radiotherapy. You know, I would go in every day and just be surrounded by old people[…] With my radiotherapy experience I was just very unprepared for the side effects because I’d been told that you know you’ve had chemo, you’ve had this massive dose of chemo, this won’t affect you, radiotherapy is easy, it’s a breeze so I went into it thinking oh ok easy peasy but I came out with a lot of side effects that really affected me.”*

## Necessity of a dedicated, compassionate project coordinator

**Fig. 1:**
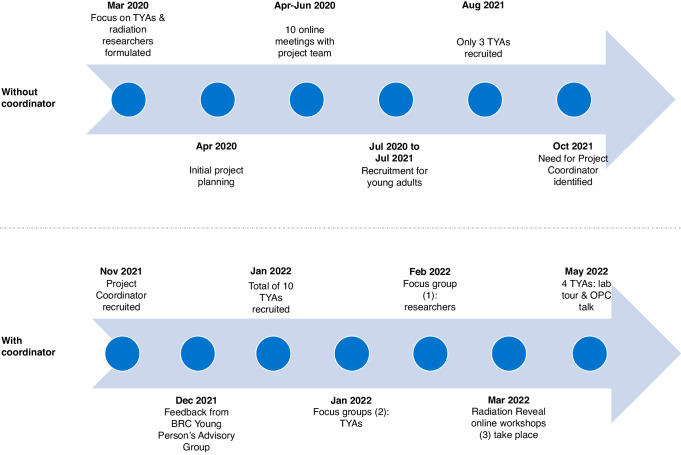
Timeline of Radiation Reveal from conception to completion, both before and with the funded project coordinator driving the project. TYA teenage and young adults with radiotherapy experience, BRC Biomedical Research Centre, OPC Oncology Professional Care annual conference.

Initial planning started in April 2020 with 10 online meetings held between the organising team consisting of researchers, university engagement managers and officers, and clinical oncologists across RadNet CoL and the Centre of the Cell (Fig. 1). Despite a real enthusiasm to carry out the project, the project team (10 people, none of whom received funding for this project) found it challenging to recruit participants; only three young people were recruited via a clinical consultant.

It was only once a freelance project coordinator was recruited through RadNet CoL that the project progressed (Fig. 1). Her professional and personal experience in this area, links to several national cancer charities, and dedicated time and focus on the project were crucial for participant recruitment, both patients and researchers, successful workshop preparation and delivery. Coming up with links to charities was considered highly valuable as these take time and strong communication skills to build up.

## Terminology and working practice

The project coordinator discussed the proposed workshops with the Biomedical Research Centre’s Young Person’s Advisory Group (healthy volunteers) in December 2021, who ranged between 17 and 25 years old. Most stated a desire to be called ‘young adults’, and the importance of ascertaining best working times and communication approaches, e.g. email, WhatsApp or telephone calls, for participants going forward. The group highlighted the need to support participants as they may find talking about their experiences of radiotherapy upsetting.

Due to the highly emotive and sensitive nature of some of the topics, many preparatory discussions were organised between PPI coordinators, heads of engagements, and lead nurses at several national charities, as well as with a psychologist at a children’s hospital, and several other professionals at NHS trusts across the UK. The project coordinator was present in all workshops and had training and experience to support patient participants acquired in their previous professional roles as a support worker for cancer patients via their experience working at several charities. A clinician was also involved.

## Recruitment

The project coordinator contacted colleagues in cancer charities who support teenagers and young adults, e.g. Young Lives versus Cancer, Shine Cancer Support, Trekstock, Teenage Cancer Trust, Teens Unite Against Cancer, and Live Through This. The aim was to 1) gain feedback on the project and 2) ask to advertise Radiation Reveal within their networks. Five charities shared the opportunity. The project was also advertised through social media and clinical contacts. Potential participants were given options of how to get in touch, e.g. by completing an online survey, email, texting, or calling the project coordinator. Once someone expressed interest, a call was arranged to discuss the project; these lasted between 15–90 minutes depending on individuals’ needs.

By January 2022, 10 young adults aged 17–25 were recruited (Fig. 1). They all lived and were treated with radiotherapy in England and had been diagnosed with rectal cancer, a brain tumour, thyroid cancer, Non-Hodgkin lymphoma, sarcoma and chordoma. Some had also received chemotherapy and immunotherapy. Seventeen people who contacted the project coordinator were aged over 25 years-old and so they were signposted to and participated in a 2-hour event for an older audience through Trekstock around radiation experiences. Ethical approval was not required for these workshops, however, the principles of informed consent still applied. As such patient participants were given full information about what they were being asked to do, had every opportunity throughout to ask questions, and an option to withdraw at any time.

Five laboratory-based CRUK RadNet CoL researchers were also recruited to the project, namely two PhD students, one Junior Group Leader, one Senior Research Fellow, and one Senior Lecturer.

## Preparatory focus groups

**Fig. 2:**
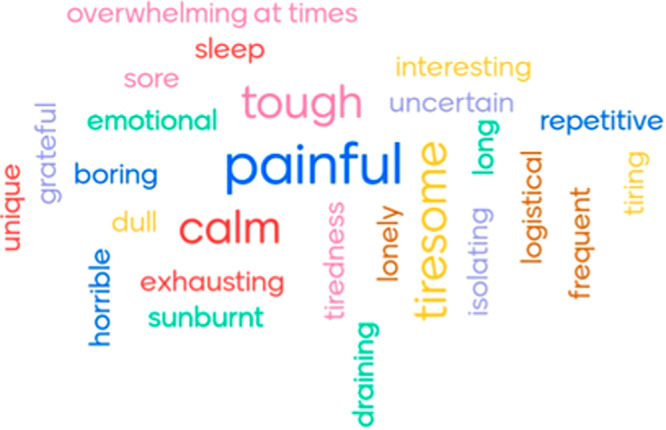
Summary of radiotherapy experience by young adults. Word cloud of responses by the 10 young adults with radiotherapy experience during the two focus groups held in January 2022 to the question ‘What word(s) would you use to describe your radiation/radiotherapy treatment?.

Two 2-hour online focus groups were run in January 2022 (Fig. 1) enabling TYAs to meet, share stories and find common ground in terms of their diagnosis and treatment experiences. From the outset, everyone was open, honest, and highly engaged. During the focus groups, we asked the young adults to describe their radiotherapy treatment (Fig. 2). We decided the best communication approach to get to know each other, connect, and arrange logistics, was a WhatsApp group.

The Centre of the Cell ran a public engagement training workshop for the researchers. Also, a one-hour focus group with researchers was conducted in February 2022 (Fig. 1) to discuss their expectations of the workshops and any concerns. For most, this would be the first time they had spoken to anyone with lived experience of cancer. Researchers said they felt removed from the patient experience and wanted to understand treatment experiences and what aspects required improvement. They were also interested in TYA’s perceptions of what research is, what type of work researchers do, and how science progresses. We also covered describing their work in an accessible way, the use of appropriate language and terminology, and sensitivities to be aware of.

The project coordinator shared short researcher biographies and photos with the young adults before workshops began; it became apparent that two participants knew each other from university but had lost touch until this project. This approach increased the depth of relationships formed between all participants from the get-go.

## Workshops

Following TYA feedback, three 2-hour workshops, facilitated by the project coordinator and the Centre of the Cell, took place on Wednesday evenings via Zoom in March 2022 (Fig. 1). Attendees included the project coordinator, a member of Centre of the Cell, the young adults and researchers, a visual artist who captured the conversations, and a clinical oncologist with expertise in both the patient treatment pathway and research. The word cloud created from answers to the question ‘What word(s) would you use to describe your radiation/radiotherapy treatment?’ (Fig. 2) provided a starting point for the workshop discussions. Following each workshop, participants completed a short reflection survey and the project coordinator checked-in with the group via WhatsApp and circulated some useful numbers for additional support should this be required.

### Workshop 1: Overview of cancer, treatments, and story sharing

**Fig. 3:**
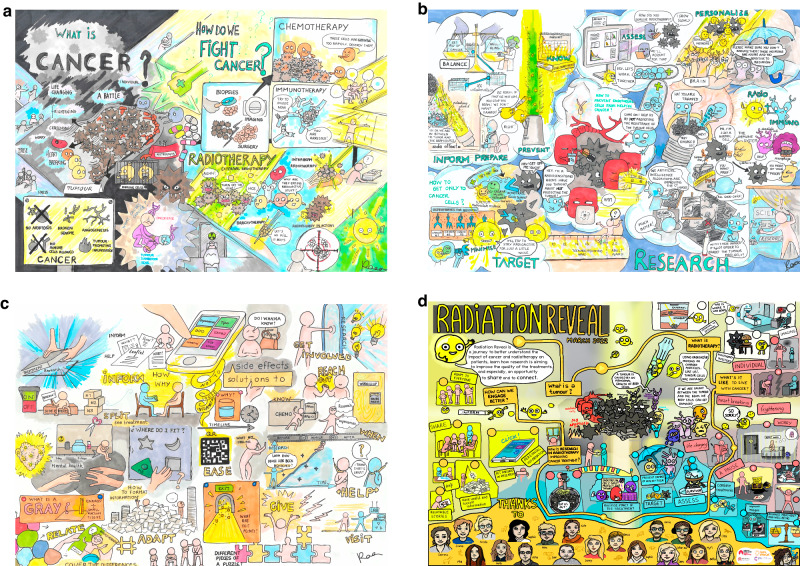
Artwork by Virginia Fernandez capturing workshops. **a**, (**b**) and (**c**) capture workshops 1, 2, and 3, respectively. **d** Artwork summarising the Radiation Reveal journey throughout the three main workshops.

The project coordinator invited TYAs to join the call a few minutes early so they could greet each other and settle before other participants arrived. An icebreaker was used as a form of introduction. A brief overview of what cancer is and how it can be treated was then provided (Fig. 3A). It was reiterated that this project was focussing on radiotherapy and TYAs were then invited to share their experience of having cancer. Everyone spoke openly and honestly. All were extremely supportive. For most, aside from our focus groups and newly-formed WhatsApp group, this was the first time they had spoken to their peers about their cancer experience. Reflections included:

*“My favourite part of the workshop was getting to chat to other patients about their experiences and answer questions from the researchers. Having the opportunity to be in a group of people with a shared experience and of similar age is fairly uncommon for me”* (from young adult)

and

“*Hearing really open and honest stories from the patients talking about their struggles with diagnosis, treatment side effects and emotions. It’s a big part of cancer that we as researchers don’t think about*.” (from researcher).

### Workshop 2: Radiotherapy, researcher and clinical oncologist talks

During the second workshop, each researcher gave a presentation (Fig. 3b) and TYAs asked insightful questions. The clinical oncologist also talked about her role and involvement in bringing research into a clinical setting.

When discussing the research and where it should head, the input from the TYAs was mostly around short- and long-term side effects. Conversations about research continued between TYAs on WhatsApp with one young person making a diagram of how researchers might treat their type of brain tumour.

Reflections included:

*“I enjoyed finding out about all the new ideas within the radiotherapy research field - feel really privileged to have got behind the scenes”* (from young adult)

and

*“The best part of this workshop was questions from the young people, really insightful hearing their views and some of their ideas!”* (from researcher).

### Workshop 3: Helping future patients

**Fig. 4:**
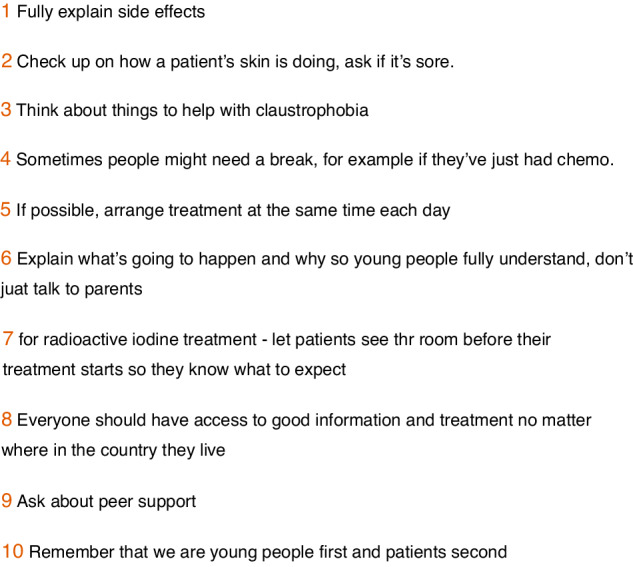
Top 10 tips created during the final workshop for medical professionals treating young adults across all radiotherapy types.

In the final workshop, TYAs described what they wish they had known before treatment and how we could help enhance the experience of future patients undergoing radiotherapy (Fig. 3c). They wanted to turn a negative experience into something positive. Discussions were had around how to make written information, e.g. patient information leaflets, more visually appealing, and creating an app for TYAs diagnosed with cancer. Many ideas were shared such as a QR code on an information postcard, instead of leaflet, with details created as an infographic rather than text. The group then came up with ten tips for healthcare professionals involved in radiotherapy to improve the experience for other young people (Fig. 4).

Two informal online catch ups were then run for the young adults as everyone was reluctant for the project to end.

## Outputs

Our project aimed to initiate discussions between young adults and radiation researchers and identify what people wish they had known about radiotherapy before or during treatment; these were achieved (Fig. 3d). One young adult participant perfectly described this:

*“Thanks everyone for great workshops; really enjoyed it… it’s like it broke down the barrier between the experts on one side and the experts on the other.”*

Other outputs included the artwork summarising each workshop and overall Radiation Reveal project (Fig. 3), peer support, lasting friendships and the creation of a new social media account and email address offering support to other young people diagnosed with thyroid cancer. Many of the young people continued their involvement in subsequent patient involvement and engagement projects.

Also, the top ten tips for healthcare professionals created together during the final workshop with all participants (Fig. 4) were disseminated at three national conferences; Oncology Professional Care 2022 and 2023, and UK Imaging and Oncology 2023, as both oral and poster presentations.

An overview of the project and the top 10 tips were shared with the Teenage and Young Adult Cancer Service at Guy’s Hospital (March 2022), and on episodes of the first therapeutic radiographer-led oncology podcast, RadChat (https://radchat.transistor.fm/episodes, episode 47 (April 2022) and Bonus Episode (October 2022)).

In May 2022, four of the young adults visited Bart’s Cancer Institute for a lab tour with the project coordinator and two of the researchers and visited Centre of the Cell for an educational science show. Finally, conversations have continued between the young adults and researchers to help guide future radiation research.

## Discussion

This creative and collaborative public engagement project brought together young adults who have had radiotherapy and radiation research. The process and outputs provide a unique view of patient experience and demonstrate the meaningful impact patient engagement and involvement can have.

Previous work [1] has explored the unique challenges that adolescent and young adult cancer survivors face. Having cancer as a teenager or young adult is very different from having it as a child or later in life. Getting a diagnosis and treatment can be challenging for many reasons. For example: if you get sick as a young adult or teen, doctors might not consider cancer as a cause. Once diagnosed and given a treatment plan, young adults may also be caught in the gap between paediatric and adult services with little peer support. We wanted to give young adults the opportunity to share their experiences, connect with peers, and find out about our radiation research and shape future radiation research.

Key themes in our workshop discussions echoed previous research. Young adults spoke about 1) being young and diagnosed with cancer, 2) feeling different to friends and 3) the need for peer support. It was clearly the first time many of the group talked to anyone of a similar age who had been diagnosed with cancer and received radiotherapy. This project adds weight to previous research [7], which highlighted the “paradox of being young and having cancer”. It is important for researchers and clinicians alike to keep listening to young adults to understand how to best meet their needs.

Key learnings were that it requires a dedicated and (com)passionate person with connections to national cancer charities to recruit patients and involve them appropriately. When designing the project, constant iterative feedback is needed not only from the charities, but also young adults both with and without radiotherapy experience. Finally, visually capturing the essence of discussions and keeping the door open beyond the workshops further enhances impact.

We felt strongly that we wanted to run the project in line with NIHR guidance [8] and reimbursed each young person for their time taking part in the project. This is one way in which people can be given recognition for the time, skillset, and expertise that they contribute to the research process even if money is not a motivating factor for participants; it certainly was not here.

We also learned important lessons when running an engagement project online. Efforts were made to ensure the young people felt comfortable. Including allowing the young adults to join the online call before the researchers, using humorous ice breakers and acting on feedback from reflective surveys at the end of each workshop. Many young people also used the WhatsApp group to communicate with the project coordinator during workshops. One possible improvement for the future is to break into smaller groups to ensure all discussions can be completed within the allotted time.

Radiation Reveal made a positive impression on all researchers. They expressed a heartening and strong personal impact from hearing the young people’s experiences with treatment and life during and after radiotherapy. The researchers said they learned a great deal about the clinical aspects of radiotherapy, having become more aware of the often dismissed gap between laboratory research and how patients experience its clinical application. According to the researchers, filling in that gap with more information may lead to more effective research. This is reflected in the following quote from a researcher:

*“I just wanted to thank you for organising the Radiation Reveal workshops which have been so insightful for me and motivating for my work. I have been speaking to other colleagues at work and many are really interested in gaining better patient/clinical perspective, as we so easily lose sight of the bigger picture.”*

However, all researchers acknowledged the limited impact of the project on their research work. All referred to pre-established research guidelines and scope, conditioned by funding and pre-approved research proposals. It is possible that factors like researcher seniority and research field also restrict the impact on research direction and it became clear that there is a need to correctly time public and patient involvement projects, i.e. pre-funding. They said, therefore, that Radiation Reveal had not yet directly informed the scientific direction of their work. Two principal investigators have since submitted funding proposals (which included plans for patient involvement activities) on topics discussed during the workshops; this shows the necessity of public engagement and involvement activities to include not only early career researchers, but principal investigators also. Simultaneously, two researchers expressed a change in their mindset while approaching research and said that a closer relationship with patients’ clinical experiences had inspired new research questions and ideas for future research, especially regarding treatment side effects. Finally, some researchers have continued cultivating a culture of patient involvement within their research activity and have since been leading independent projects building from Radiation Reveal.

The project was praised for creating an environment that favoured honesty and openness; one of the researchers attributed this to forming a group within a similar age range. Researchers saw Radiation Reveal as a step towards better young adult care, aimed at important challenges still to be overcome, such as helping professionals understand the patients’ point of view; better informing patients; getting information across to clinicians, patients, and their families, and putting patients in contact with each other.

Since this project, all researchers have continued in other involvement and engagement projects, some of these with young adults from Radiation Reveal. By making use of peoples’ knowledge, lived experience, and networks, public involvement helps make research more relevant to the end-users and ultimately leads to better services, treatments, and care [9]. The value and benefits of public and patient involvement in research have been well documented but there can be challenges both in recruiting younger participants and moving towards involving patients, particularly around lab-based research. Radiation Reveal started as an engagement project but as the project continued, relationships were built and the young adults had more involvement in research, not just with the researchers involved in this project but also through other patient involvement groups. Researchers valued this experience and have since initiated further projects to involve patients in their research.

With an increasing request from funders to plan and carry out public engagement as part of the funding and research cycles, we hope to have provided others with useful ideas to develop their own meaningful patient and public involvement and engagement activities.

## Conclusion

Radiation Reveal had short- and long-term positive impacts on the young adults who previously experienced radiotherapy and researchers who took part. Knowledge from the project was disseminated to the clinical community. We hope to inform and inspire researchers, including those involved in basic science, to run similar projects and help project the patient voice in all we do.
